# Potential biomarkers: Identifying powerful tumor specific T cells in adoptive cellular therapy

**DOI:** 10.3389/fimmu.2022.1003626

**Published:** 2022-11-14

**Authors:** Wu Ge, Yuqian Dong, Yao Deng, Lujuan Chen, Juan Chen, Muqi Liu, Jianmin Wu, Wei Wang, Xiaoqian Ma

**Affiliations:** ^1^ Cell Transplantation and Gene Therapy Institute, The Third Xiangya Hospital, Central South University, Changsha, China; ^2^ Department of Radiology, The Third Xiangya Hospital of Central South University, Changsha, China

**Keywords:** tumor infiltration lymphocyte, tumor specific T cell, biomarker, adoptive cellular therapy, cancer treatment

## Abstract

Tumor-specific T cells (TSTs) are essential components for the success of personalized tumor-infiltrating lymphocyte (TIL)-based adoptive cellular therapy (ACT). Therefore, the selection of a common biomarker for screening TSTs in different tumor types, followed by *ex vivo* expansion to clinical number levels can generate the greatest therapeutic effect. However, studies on shared biomarkers for TSTs have not been realized yet. The present review summarizes the similarities and differences of a number of biomarkers for TSTs in several tumor types studied in the last 5 years, and the advantages of combining biomarkers. In addition, the review discusses the possible shortcomings of current biomarkers and highlights strategies to identify TSTs accurately using intercellular interactions. Finally, the development of TSTs in personalized TIL-based ACT for broader clinical applications is explored.

## Introduction

The abundance of antitumor immune cells in tumor-infiltrating lymphocytes (TILs) is positively associated with good prognosis in most tumor types and serves an important role in tumor control (1–4). Analyses of the components of TILs have revealed that the T cells were in different developmental states in the tumor, such as effector T cells, memory T cells, incompetent T cells and exhausted T cells, while only T cells in an activated state were capable of producing actual antitumor effects (5). In fact, activated T cells were classified as tumor-specific T cells [TSTs; recognizing tumor-specific antigens (TSAs), such as cancer/testis antigen 1B and melan-A (MART-1)] and bystander T cells (only recognizing cancer-independent antigens, such as Epstein-Barr virus, human cytomegalovirus or influenza virus antigens) (6, 7). Successful antitumor immune responses after immune checkpoint blockade therapy were considered to require reactivation and clonal expansion of TSTs (8–10). Therefore, efforts have been made to isolate a TST population from TILs for use in adoptive cellular therapy (ACT), although isolating and expanding enough TSTs is another major challenge.

In recent years, the detection and sorting of TSTs using different bioinformatics techniques has stimulated great interest among researchers in identifying biomarkers, and the identification of biomarkers was of great significance in enriching the tumor-specific TIL population, studying endogenous antitumor immune mechanisms, and identifying antigen-specific T-cell receptors (TCRs) or new mutant antigens (11–14). In addition, the observation that lack of clear tumor-specific antigens was associated with the deficiency of TST responses in some tumor types, such as ovarian cancer (15), further emphasizes the necessity of biomarker testing of T cells.

**Figure 1:**
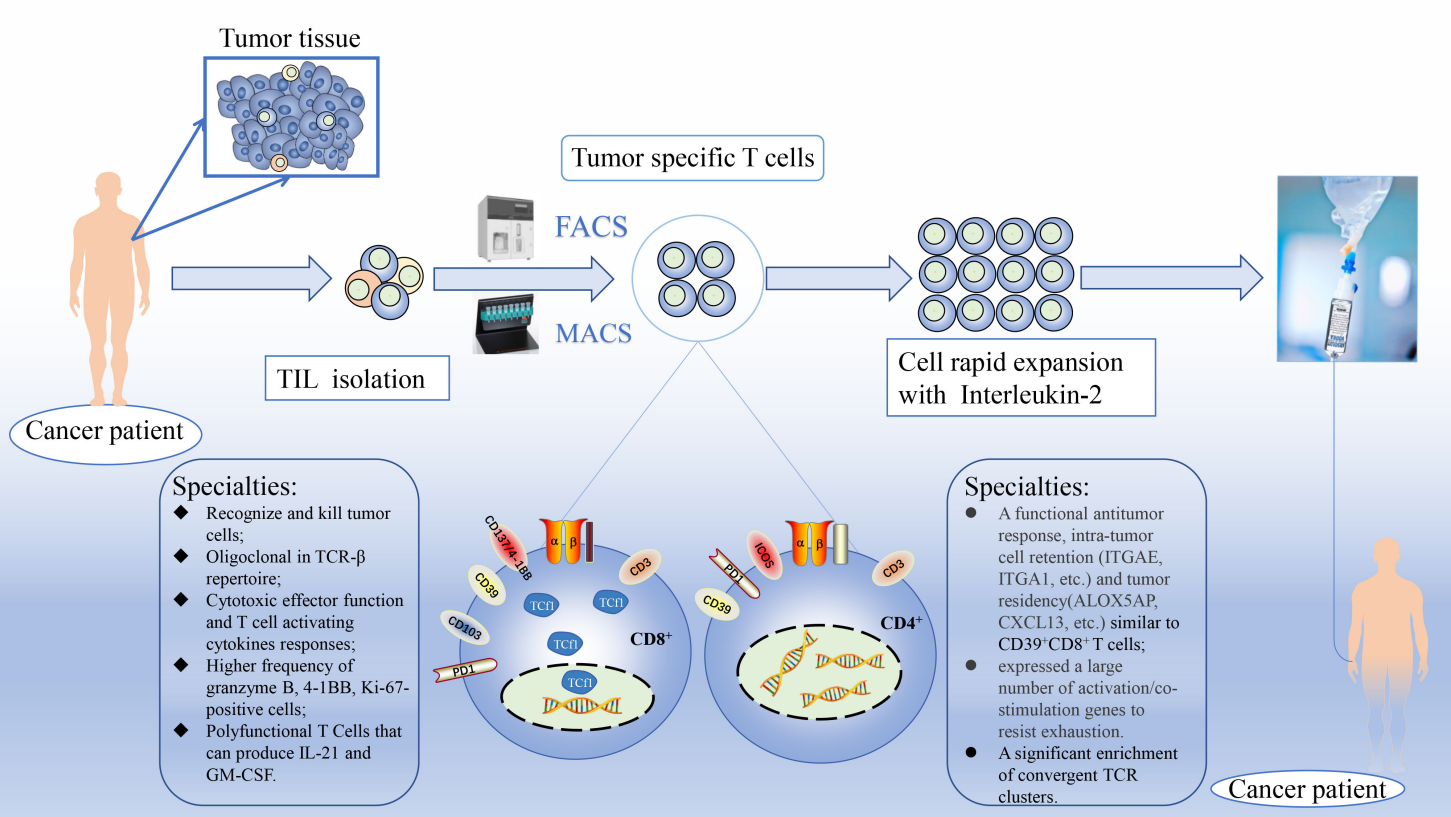
Schematic diagram of personalized enrichment process for TIL therapy. After resect tumor from the patient, the tumor is digested into small fragments or single cell suspensions. Then, sorted out tumor-specific T cells from TIL based on known biomarkers accurately and expanded them in culture with IL-2 rapidly. Finally, TST cultures are expanded to clinically relevant levels and reinfused back into the patient. FACS, fluorescence-activated cell sorting; MACS, magnetic bead-activated cell sorting; Tcf1, T cell factor 1.

Among the biomarkers of TSTs initially identified in different tumor types, the immunomodulatory suppressor receptor programmed cell death receptor 1(*PDCD1*), the chronic antigen activation marker CD39 (*ENPD1*), the tissue-resident marker CD103 (*ITGAE*) and the costimulatory receptor CD137 (4-1BB, *TNFRSF9*) were mainly targeted as objects of interest (16–19). CD8^+^ T cells are recognized as a population with antitumor effects, while CD4^+^ T cells have the advantage of enhancing the recruitment and effector functions of tumor-specific CD8^+^ T cells and activating natural killer cells in tumors, which has prompted investigators to screen for TSTs based on both subpopulations combined and individually (**Figure 1**). At present, there is no clear shared biomarker for TSTs in different tumor types. The present review summarizes several of the most popular biomarkers being studied and assays with potential for clinical biomarker detection in the last 5 years, which is important for the development and clinical application of personalized TIL-based ACT by identifying the common characteristics of different biomarkers to determine the most specific T cell population in different tumor types.

## Individual molecular markers

### PD-1^+^CD8^+^ T cells

Programmed cell death receptor 1 (PD-1) is an important immunosuppressive molecule originally found to be expressed on the surface of T cells, which is highly expressed on all activated T cells. PD-1 induces programmed T-cell death by binding to programmed cell death ligand 1/2 (PD-L1/2) and transducing T-cell failure signals. Therefore, PD-1 has been identified as a molecule that negatively regulates the antitumor immune response of T cells (20). Furthermore, the higher the proportion of PD-1 expression on T cells was, the more terminally exhausted T cells tended to be, and the weaker their proliferative capacity and ability to produce cytotoxic cytokines were, resulting in their impaired or inhibited function in controlling tumor growth (21). High PD-1 expression on the surface of activated T cells in TILs is exploited as a biomarker for the identification of TSTs. Early studies revealed that there was a markedly higher proportion of PD-1^+^CD8^+^ T cells than PD-1^−^CD8^+^ T cells in melanoma-infiltrating lymphocytes. After sorting and expansion *in vitro*, the former contained a higher proportion of MART-1-specific T cells, although this cluster was accompanied by impaired effector function, which tentatively demonstrated that tumor-specific CD8^+^ T cells were predominantly PD-1^+^ T cells (21, 22). Gros (16) et al. reported that the PD-1^+^CD8^+^ TIL population recognized and killed autologous tumor cells compared with their negative controls in six tissues from patients with metastatic melanoma (MM), and the PD-1^+^CD8^+^ TIL population with specific TCRβ clonotypes contained clonotypes targeting mutant antigens and reserved the ability to recognize autologous tumor cells, showed highly monoclonal expansion. These findings demonstrated that PD-1 expression on CD8^+^ TILs also accurately identified a clonally expanded TST repertoire. Further analysis of the TCR-β profiles of both PD-1^+^ and PD-1^−^ populations in CD8^+^ T cells revealed that there was hardly any overlap in the TCR profiles of PD-1^+^ and PD-1^−^CD8^+^ T cell subpopulations in at least two human tumor tissues, colorectal cancer (CRC) and breast cancer, thus, it can be concluded that the intrinsic properties of the PD-1^+^ population determine its ability to recognize tumor antigens specifically (23). A comparison of PD-1^+^CD8^+^ TIL and PD-1−CD8^+^ TIL expansion products in mouse solid tumor models (melanoma and colon cancer) and multiple myeloma models revealed that TSTs existed exclusively in PD-1^+^CD8^+^ TIL progeny and inhibited tumor progression. Furthermore, combination with anti-PD-L1 treatment further enhanced the efficacy of TIL therapy, which provided the basis for preclinical experiments on PD-1^+^CD8^+^ TIL expansion in ACT (24). High PD-1 expression by TSTs in the peripheral lymphoid organ, the spleen, has also recently been revealed, and peripheral PD-1^+^CD8^+^ TSTs were reported to be positively associated with the secretion of IFN-γ in lymphoid tissue after vaccination (25). Likewise, the similarity in tumor antigen specificity and TCR repertoire of PD-1^+^CD8^+^ T cells in both circulating peripheral blood and TILs implies that circulating PD-1^+^CD8^+^ T lymphocytes could be a window to provide access to TSTs, and thus, PD-1 expression identified diverse patient-specific antitumor T cell responses in the peripheral blood, providing a novel non-invasive strategy for developing personalized therapies for cancer using neoantigen-reactive lymphocytes or TCR-T cells (26–28). In addition to demonstrating the specificity of PD-1^+^CD8^+^ TSTs sorted in freshly extracted TILs, a study analyzed the dynamic changes of tumor-specific CD8^+^ T cells in patients who received CD8^+^ TIL-transfer therapy and were observed for up to 1 year, and the investigators found that the persisting T cell subpopulations after tumor-specific CD8^+^ TIL treatment were mostly multifunctional, with a stable partially differentiated phenotype and high expression levels of PD-1 (29). The conclusion suggested that PD-1 can be a stable biomarker for TSTs. Therefore, the aforementioned extensive studies demonstrate the superiority of PD-1 as an individual marker in identifying CD8^+^ TSTs.

### CD39^+^ T cells

CD39 is an extracellular enzyme encoded by the ectonucleoside triphosphate di phosphohydrolase 1 gene, and early studies found that CD39 participated in a cascade reaction with CD73 for the conversion of ATP to ADP and cAMP, ultimately leading to the production of adenosine, a molecule that produces extracellular immunosuppressive effects (30–32). CD39 expression in T cells has been described as a marker of exhaustion. Early studies of CD39^+^CD8^+^ T cells investigated the comparison with their counterpart, CD39−CD8^+^ T cells, in TILs or metastatic lymph nodes from 33 untreated patients with breast cancer and 4 patients with melanoma, as well as in mouse tumor models (breast cancer and melanoma), the former expressed relatively low levels of TNF-α, IFN-γ and IL-2, and were negatively associated with the secretion of granzyme B and perforin. Additionally, CD39 expression was accompanied by the expression of co-inhibitory receptors (i.e., lymphocyte activating 3, T cell immunoreceptor with Ig and ITIM domains, PD-1, T-cell immunoglobulin mucin family member 3, and 2B4) on T cells and was associated with tumor growth (33, 34). However, in 2018, a study published in Nature reported that the direct distinction between TSTs and bystander T cells within TILs was CD39 expression, which demonstrated that CD39^+^CD8^+^ TILs were a population that could recognize tumor neoantigens specifically (7). Based on the publication of the novel insight, a study using whole-exome sequencing algorithms to analyze high-affinity neoantigens (HANs) in tumor tissues from 56 patients with hepatocellular carcinoma revealed that HANs were associated with improved overall survival and the frequency of CD39^+^CD8^+^ TILs in patients with hepatocellular carcinoma, and identified HANs− specific CD8^+^ T cells in CD39^+^CD8^+^ TILs, which suggested that HANs triggered antitumor activity by activating tumor-specific CD39^+^CD8^+^ T cells, and CD39 could serve as a reliable marker for identifying TSTs (35). During the same year, this team demonstrated that CD39^+^HBVs− CAR T cells and CD39^+^ autologous tumor-specific CD8^+^ T cells induced more apoptosis of tumor cells in the hepatocellular carcinoid organ using CD39 as a marker, which was concluded based on comparisons with the CD39−CD8^+^ T cell subpopulation (36). In addition, a study of clinical staging of patients with bladder cancer (n=46) revealed that CD39 expression in CD4^+^/CD8^+^ T cells was markedly associated with tumor pathological T stage and tumor histology, and CD39^+^CD8^+^ T cells presented more potent tumor killing effects by producing higher levels of IFN-γ than other T cell populations (37).

Most recently, Liu et al. (38) revealed that tumor-specific CD4^+^ T cells differentiated into T helper 1 cells and CD4^-^ T cells, in which CD4^-^ T cells are critical for controlling established tumor metastasis and tumor specific-CD4^+^ T cells have a synergistic therapeutic effect with PD-L1 blockade. CD39 could alternatively be applied as a marker to identify a population of CD4^+^ TSTs in three human tissue squamous carcinomas (cervix, vulva and oropharynx). Single-cell RNA sequencing of the CD39^+^CD4^+^ conventional T cell cluster revealed the features associated with a functional antitumor response, intra-tumor cell retention (*ITGAE*, integrin subunit α1, etc.) and tumor residency [arachidonate 5-lipoxygenase, C-X-C motif chemokine ligand 13 (*CXCL13*), etc.]. CD39^+^CD4^+^ TSTs expressed a highly exhausted phenotype but simultaneously expressed a large number of activation/co-stimulation genes that resisted exhaustion to expand *in vitro* in response to autologous tumor antigens (39). As supported in another high-dimensional profiling study of CD4^+^ TILs in human cancer tissues, CD39 expression could be used to distinguish tumor-specific CD4^+^ T cells from diverse CD4^+^ TILs (e.g., regulatory T cells and bystander CD4^+^ T cells) (40).

At present, studies have validated the specific antitumor function of CD39^+^CD8^+^ T cells in non-small cell lung cancer (NSCLC) tumor tissues, which confirmed that CD39^+^CD8^+^ T cells could be the predictor of prognosis in patients with NSCLC after anti-PD-1/PD-L1 therapy (41) and defined the protective prognostic role of CD39^high^ tissue memory CD8^+^ T cells in luminal-like breast cancer (42). Beyond this, in the latest study on better biomarkers of tumor-specific CD8^+^ T cells in the peripheral blood circulation, it was determined that three candidate biomarkers, including CD39, might compensate for the low sensitivity of PD-1 that served as a previous marker by single-cell RNA and TCR sequencing, at least in the blood of MC38 tumor-bearing mice and patients with melanoma (43). Therefore, as a chronic local antigen activation marker, the efficacy of CD39 to identify tumor-specific effector T cells deserves in-depth investigation.

### CD103^+^CD8^+^ T cells

CD103 is an integrin molecule, which is expressed mainly on intraepithelial T cells and TILs, and is involved in the migration and residency of T cells in tissues. The latest newly defined tissue-resident memory T cells (TRMs), whose main surface markers are CD103, CD69 and CD49a, are classified as a cluster of memory T cells different from central memory T cells and effector memory T cells, and this population persists in tumor tissues and no longer participates in T cell recirculation (44–47). Intratumor CD8^+^ TRMs are a subpopulation with identified antitumor functions, in which CD103 is predominantly expressed on the surface, and intra-tumor CD103^+^ TILs might serve as a prognostic marker for patients with urothelial cell carcinoma of the bladder (48). Therefore, studies on the role of CD103 are mainly based on the CD8^+^ TRM population. In earlier research, investigators revealed that CD103^+^CD8^+^ TRMs expressed high levels of granzyme A, granzyme B, perforin, IFN-γ and TNF-α, as well as the degranulation marker CD107a and the proliferation marker Ki-67 according to the analysis of cytotoxic function-related effector cytokines in *ex vivo* experiments, suggesting that CD103 was associated with cytotoxic activity in CD8^+^ T cells (49–52). Since then, several studies have reported that CD103^+^CD8^+^ TRMs isolated from TILs of various tumor types were a good prognostic marker for patient survival. Indeed, in immunohistochemistry cohort studies of patients with breast cancer (n=424) and endometrial adenocarcinoma (n=305), the high levels of CD103^+^ TIL infiltration predicted a good prognosis and prolonged survival (53, 54). In a single-cell analysis of breast cancer-infiltrating T cells, CD8^+^ TRMs also showed an association with a favorable prognosis in early-stage triple-negative breast cancer (55). In bladder uroepithelial carcinoma, CD103^+^ TILs have been identified as a predictive marker for overall survival and good prognosis for recurrence-free survival, and the study showed that tumor volume was negatively associated with CD103^+^ TIL infiltration density (48). A positive association between TRM and good prognosis of hepatocellular carcinoma was also confirmed in a multidimensional analysis of hepatitis B virus-associated hepatocellular carcinoma (56). Analysis of CD103^+^CD8^+^ TRMs in a study of NSCLC revealed that their components contained TSTs that recognized TSAs, a subpopulation associated with improved survival and increased intraepithelial lymphocyte infiltration in patients with early-stage NSCLC (57). The higher density of intra-tumoral and stromal CD103^+^CD8^+^ TIL cells in oral cancer also predicted an improved prognostic performance, and CD103^+^CD8^+^ TILs had the same phenotype as TRMs (58). At present, studies on CD103 as a marker to identify tumor reactive T cells in TILs are getting more and more advanced. On the one hand, given that multiple suppressive immune checkpoint molecules [e.g., PD-1, cytotoxic T-lymphocyte associated protein 4 (CTLA-4) and TIM-3] are highly expressed on the surface of CD103^+^CD8^+^ TRMs (59, 60), several studies have identified CD103^+^CD8^+^ TRMs as the population serving major antitumor efficacy in patients with different tumor types treated with anti-PD-1/PD-L1 immune checkpoint therapy. It has been demonstrated that a low proportion of CD103^+^CD8^+^ TRMs was strongly associated with immunotherapy failure in patients with different tumor types, such as esophageal squamous cell carcinoma, lung cancer and melanoma (18, 61–63), which showed indirectly that the role of CD103^+^CD8^+^ TRM subsets in immune checkpoint therapy cannot be ignored. On the other hand, the density of CD103^+^CD8^+^ TRMs increased during anti-PD-1 treatment of responsive patients with lung cancer, with the bursting proliferative activity and cytotoxicity (e.g., granzyme B), oligoclonal expansion of the TCR-β profile clonotype and upregulated expression of Aiolos, phosphorylated STAT-3 and IL-17, which were key regulators of T helper 17 cell differentiation (61). Recently, direct evidence was obtained in the transcriptomic analysis of mutation-associated neoantigens (MANA) in lung cancer treated with anti-PD-1 therapy; ~90% of MANA-specific CD8^+^ T cells had the same signature transcriptional program as TRMs, which indicated the presence of TSTs in the TRM population, and CD103, the marker defining TRMs, might also act as a marker for identifying TSTs (64). In addition to the CD103^+^CD8^+^ TRM population, Abd Hamid (65) et al. found that cytotoxic CD8^+^ T cells (CTLs) maintained CD103 expression by self-secreting TGFβ1 in an activated form, which improved cellular TCR sensitivity as well as cell migration, contributing to the rapid recognition of autologous tumor cell surface antigens and toxic efficacy of CD103^+^ tumor-specific CTLs in killing tumor cells; however, this population was more susceptible to apoptosis during prolonged exposure to the cancer microenvironment (i.e., the proportion of CD103^+^ tumor-specific CTLs decreased as the tumor grew). Meanwhile, CD103^+^ tumor-specific CTLs were found to have a markedly higher basal glycolytic rate and elevated maximal glycolytic capacity in terms of cellular energy metabolism, which also contributed to the induction of more effective and faster antitumor efficacy. Therefore, TSTs, which are a subpopulation of CD103^+^CD8^+^ TILs, are essential in the antitumor immune response and favorable prognosis.

### CD137^+^CD8^+^ T cells

CD137 is a member of the tumor necrosis factor receptor family(*TNFR*) (66) and is expressed on the surface of activated T cells. Binding of CD137 on T cells with its ligand CD137L that expressed on dendritic cells (DCs), activated B cells and macrophages leads to markedly increased T cell proliferation, differentiation and production of effector cytokines. Therefore, CD137 has been described as a surface marker for the identification of activated T cells (67–69). It has been suggested that anti-CD137 treatment could also, promote T cell proliferation independently of CD28 (a costimulatory signaling for T cell activation) and their IL-2 production had similar extent of activation to that shown by the treatment with a combination of anti-CD3 and anti-CD28. However, CD137 could replace the effect of CD28 signaling on T cell activation in certain degree only in the presence of consistent antigenic stimulation (70, 71). Furthermore, it has been demonstrated that CD137L could stimulate human CD28− T cells, leading to the differentiation and proliferation of their cell subpopulation, with subsequent increased expression of cellular inflammatory cytokines IFN-γ, granzyme A and perforin, which ultimately enhanced their cytotoxic effector functions and the expression of the anti-apoptotic protein Bcl-X (72). *In vitro* enrichment of antigen-specific T cells by three different methods (CD137, CD107a and tetramers) using HLA-A24-restricted CMV pp65 and EBV BRLF1 epitopes as model antigens revealed that CD137-based isolation of antigen-stimulated CD8^+^ T cells was equivalent to tetramer-based sorting in terms of purity, and superior to the other two methods in terms of subsequent cell expansion (73). As such, tumor-specific effector T cells were successfully isolated from peripheral blood based on the early expression of CD137 on most activated antigen-specific CD8^+^ T cells without prior knowledge of the specific immunogenic epitopes or HLA-restricted elements identified (74). Similarly, TSTs were present in the population of CD8^+^ T cells sorted on the basis of CD137 in fresh ovarian cancer tissues, and CD137^+^CD8^+^ T cells have been demonstrated to possess specific cell killing ability against autologous tumor cells *in vitro* and to inhibit the progression of tumors in both melanoma and ovarian cancer NSG mouse models compared with PD-1^+^ or PD-1−CD137−CD8^+^ T cells (75). By enriching TILs based on CD137 upregulation of TSTs after *in vitro* stimulation, 27 tumor-specific TCRs were successfully isolated from 6 patients in a clinical study that identified 14 neoantigens expressed by autologous tumor cells, suggesting that the potential of peripheral blood lymphocytes to become true TSTs by introducing recognized TCRs might be realized in the future (17). After expansion of CD137^+^ TILs isolated using the magnetic bead sorting technique *in vitro*, it was found that the expanded cell population showed markedly increased antitumor reactivity and was enriched with T cells that recognized neoantigens as well as shared tumor antigens (76). Interestingly, the expanded products of CD137^+^ T cells *in vitro* secreted multiple cellular cytokines, including IFN-γ, TNF-α and IL-2, after co-culture with a mixture of preferentially expressed antigen in melanoma-derived peptides, a specific tumor antigen upregulated in melanoma obtained from the peripheral blood of healthy donors (77). Recently, a systematic analysis of four biomarkers, PD-1, CD39, CD103 and CD137, in ovarian cancer TILs found that PD-1^+^, CD39^+^ and CD103^+^ TILs all contained a subpopulation of CD137^+^ T cells and CD137^+^ TILs highly co-expressed the three previous markers, with the highest expression of major histocompatibility complex (MHC)-dependent IFN-γ and other cellular effector cytokines (78). In addition, this subpopulation also uniquely co-expressed the costimulatory molecule CD28, suggesting that CD137^+^ TILs can recover stronger antitumor potential through combined immune checkpoint blockade (79). Thus, we tentatively hypothesized that CD137 had superior properties as a natural biomarker for identifying tumor-specific effector T cells. In three types of human cancer samples (23 MM, 1 ovarian cancer and 1 sarcoma sample), researchers have recently proposed that application of the combination assays of total intracellular CD137 expression, tumor reactive cytokines (IFN-γ and TNF-α) and scRNA analysis data could obtain the rapid and accurate *in vitro* identification of a larger proportion of TSTs, such a cell population not only had the specificity to recognize tumor neoantigens but also had immediate tumor responsiveness. Furthermore, *in situ* detection of the corresponding genes, TNFRSF9, IFNG and TNF, may also be considered as a strategy to rapidly and efficiently identify the functional characteristics of most tumor-specific TILs based on scRNA sequencing data (80). However, TIL expansion *in vitro* from 16 cases of renal cell carcinoma revealed that, although TILs cultured with autologous tumor single cell suspensions could express a high percentage of CD137, only a small percentage of these cells expressed IFN-γ, TNFα and IL-2 detected by flow cytometry, suggesting that the tumor-specific CD137^+^ T cell subpopulation might lack tumor responsiveness to some extent (81). In summary, CD137 has the potential to be a natural biomarker for sorting a subset of tumor-specific reactive T cells *in vitro*, but one also needs to pay attention to possible mechanisms of secretion of dysfunctional cellular effector cytokines by TSTs in different tumor types (**Table 1**).

**Table 1:** Individual molecular markers.

Biomarkers of TST	Superiority	Tumor types	Animal experiments	Clinical trials	Reference
PD1^+^CD8^+^ T cells	• Ability to recognize autologous tumor cell lines or human tumor cells with IFN-γ secretion and 4-1BB up-regulation. • Contains a percentage of tumor neoantigen-specific T cells(MART-1、CDKN2Amut peptide HLA-A*1101、OVA、M8 etc.) • Unique TCRβ clonotypes.	1) Melanoma and Metastatic melanoma 2) NSCLC 3) colorectal cancer and CRLM 4) Gastrointestinal cancer 5) Renal cell carcinoma 6) Mouse tumor cell lines(B16-OVA、MC38、ID8、5TGM1)	Melanoma Colon Cancer Myeloma Multiforme	NCT00937625(MM) NCT03215810	(16, 21–24, 26–29)
CD39^+^CD8^+^ T cells	• Enriched in expression of genes related to cell proliferation、functional antitumor response、cell retention and tumor residency. • A skewed and reduced diversity of TCR sequence diversity. • Identify High affinity neoantigens specific CD8^+^ T cell. • Induce more apoptosis of autologous tumor cells compared with its negative control;	1) Melanoma &Metastatic melanoma 2) Ovarian cancer 3) NSCLC 4) Colorectal cancer&CRLM 5) Hepatocellular carcinoma 6) Breast cancer	Hepatocellular carcinoma(PDX) Bladder cancer	N/A	(7, 35–37, 40–42)
CD39^+^CD4^+^ T cells	• A functional antitumor response, intra-tumor cell retention (ITGAE, ITGA1, etc.) and tumor residency (ALOX5AP, CXCL13, etc.) similar to CD39^+^CD8^+^T cells. • Expressed activation/co-stimulation genes to resist exhaustion.	1) Squamous cell carcinoma(cervix, vulva, oropharynx) 2) Lung cancer 3) Colorectal cancer	N/A	N/A	(38, 39)
CD103^+^CD8^+^ T cells	• Actively proliferated in the tumor milieu and displayed enhanced production of cytotoxic molecules. • State of non-terminal differentiation effector memory cells. • More oligoclonal in TCR-βrepertoire. • Produce IFN-γ and IL-17 specific to Tc17 cells. • Elevated energetic potential and faster migration capacity • Improved TCR antigen sensitivity.	1) Melanoma &Metastatic melanoma 2) NSCLC 3) Colorectal cancer&CRLM 4) Hepatocellular carcinoma 5) Breast cancer 6) Cervical cancer 7) Endometrial adenocarcinoma 8) Oral cancer 9) Esophageal squamous cell carcinoma	N/A	N/A	(18, 48–51, 56, 57, 60, 61, 63, 64)
CD137^+^CD8^+^ T cells	• Recently activated cells by TCR engagement and signaling. • Upregulate survival genes, enhance cell division, induce cytokine production, and prevent activation induced cell death of T cells by co-stimulatory signaling. • Enrichment for a T-cell–inflamed gene module representing active IFN-γ response, cytotoxic effector function, and T-cell–activating cytokines responses to anti–PD-1 therapy.	1) Melanoma &Metastatic melanoma 2) Ovarian cancer 3) Hepatocellular carcinoma 4) Renal cell carcinoma	Melanoma Ovarian cancer	NCT02111863	(17, 75–80)

TST, Tumor-Specific T cells.N/A, Not Applicable.

## Combined molecular markers

To improve tumor reactivity of sorted TIL products in various solid tumors, several researchers have proposed the combination of identified markers to determine tumor-specific effector T cells rather than single molecules to obtain a subpopulation of TILs that recognize tumor antigens more accurately, promoting the widespread clinical application of personalized TIL-based ACT. Therefore, it is meaningful to summarize representative research findings constantly (**Table 2**).

**Table 2:** Combined molecular markers.

Biomarkers of TST	Superiority	Tumor types	Animal experiments	Clinical trials	Reference
CD39^+^PD-1^+^ T cells	• Mediate metastatic cancer cells dormancy through cell cycle arrest by IFN-γ and TNF-α. • A significant enrichment of convergent TCR clusters. • Enriched with activated HLA-DR^+^ and ICOS^+^ and proliferating KI67^+^ cells. • A high proportion of HPV-specific T cells.	1) Breast cancer	Breast cancer(4T1、4T07)	N/A	(81–84)
CD39^+^CD103^+^CD8^+^ T cells	• More oligoclonal compared to SP or DN CD8^+^ TILs. • Recognize and kill higher proportion of autologous tumor cells. • Higher frequency of granzyme B、4-1BB、Ki-67-positive cells. • PMA-responsive genes and mitochondrial genes Keep stable. • Polyfunctional T Cells that can Produce IL-21 and GM-CSF.	1) Melanoma & Metastatic melanoma 2) Ovarian cancer 3) NSCLC 4) Colorectal cancer & CRLM 5) Head and Neck squamous cell carcinoma 6) High-Grade Endometrial Cancer	N/A	N/A	(19, 85–88)
CD39^+^CD103^+^PD-1^+^CD8^+^ T cells	• Highly activated, reduced TCR diversity and expressed genes involved in both cytolytic and humoral immunity. • Upregulate expression of CXCL13 for coordinated antitumor B-cell responses.	1) Ovarian cancer	N/A	N/A	(89)
PD-1^+^CD137^+^CD8^+^ T cells	• Enriched with highly activated tumor-reactive T cell.	1) Ovarian cancer	N/A	N/A	(74, 90, 91)
PD-1^+^ICOS^+^ T cells	• Showed a tissue-resident memory phenotype with a oligoclonal expansion of their TCR repertoire • Recognize both tumor-associated antigens and tumor-specific neoantigens	1) Human head and neck squamous cell carcinoma 2) Colorectal cancer	N/A	N/A	(92, 93)
Tcf1^+^PD-1^+^CD8^+^T cells	• High potential to proliferation、self-renew and T cell persistence. Λ Persistence in controlling tumor growth.	1) Melanoma & Metastatic melanoma 2) Hepatocellular carcinoma 3) Renal cell carcinoma 4) gastric cancer	Mouse tumor cell line(B16、MC38)	N/A	(94–101)
PD-1^+^Bio*^+^CD8^+^T cells	• Identifying Tumor Specific Antigens. • Containing stem-like Characteristics population.	1) Mouse tumor cell line(B16、B16-OVA、E0771、MC38)	N/A	N/A	(102)

*Bio represents a biotin assay rather than a biomarker. N/A, Not Applicable.

### CD39^+^PD-1^+^ T cells

A study of pulmonary metastatic dormancy in patients with breast cancer revealed that adoptive transferred monoclonal CD39^+^PD-1^+^CD8^+^ T cells prevented distant metastases successively, although the cancer cells were not cleared completely. Notably, CD39^+^PD-1^+^CD8^+^ T cells from the primary tumor and metastatic sites rather than overall CD8^+^ T cells mediated pulmonary metastatic dormancy, raising the possibility that this cell subpopulation might have better tumor specificity and was positively associated with increased disease-free survival in patients undergoing breast cancer resection (82). A Korean research team reported that CD39^+^PD-1^high^CD8^+^ T cells were highly expressed in epithelial ovarian cancer primary tissues and metastatic sites (n=65). Not only was high PD-1 expression associated with a highly exhausted phenotype, but it was also associated with high activation of this subpopulation and tumor specificity (83). Based on evidence from a publicly available database of melanoma tumor antigen-specific TCR sequences, a previous study determined TCR clonotype clusters and found a marked enrichment of convergent TCR clusters in the CD39^+^PD-1^+^ subpopulation of CD4^+^ and CD8^+^ TILs (84). In addition to defining TSTs using CD39^+^PD-1^+^ in human cancer tissues, researchers also focused on circulating CD39^+^PD-1^+^CD4^+^ T cells in patients with human papillomavirus (HPV)-induced tumors, a cell subpopulation enriched with activated HLA-DR^+^ and inducible T cell costimulator (ICOS)^+^ and proliferating KI67^+^ cells in the peripheral circulation, as well as a high proportion of HPV-specific T cells. Overall, the proportion of circulating CD39^+^PD-1^+^CD4^+^ T cells in patients with HPV-induced tumors treated by immune checkpoint blockade may serve as a predictor of clinical response (85).

### CD39^+^CD103^+^CD8^+^ T cells

In 2018, Duhen (19) et al. reported that CD39^+^CD103^+^CD8^+^ TILs in the primary and metastatic sites of six malignant solid tumors had a unique TCR profile and killed autologous tumor cells effectively in an MHC-I-dependent manner *in vitro*, which demonstrated that this subpopulation was enriched in tumor-specific effector T cells. Furthermore, the number of infiltrating CD39^+^CD103^+^CD8^+^ T cells in human head and neck squamous cell carcinoma (HNSCC) was positively associated with a good survival prognosis. In addition, auto-neoantigen-specific T cells could be detected in the CD39^+^CD103^+^CD8^+^ TILs in 3 of 7 patients with low mutation burden CRC through whole-exome and transcriptome sequencing of inferred neo-antigenic epitopes from tumor and normal tissues (86). Transcriptional activity and transcript stability of CD39^+^CD103^+^CD8^+^ TRMs *in situ* in human high-grade endometrial cancer indicated that this cell subpopulation had favorable transcriptional activity and expressed tissue-resident transcriptional profiles in the resting state, and the expression levels of markers of T cell activation were upregulated, and cytolytic activity and cytokine production were increased in the activated state. These results revealed that CD39^+^CD103^+^CD8^+^ TRMs in high-grade endometrial cancer were a multifunctional T cell population with a reactive response pool that included a high degree of tumor responsiveness. Furthermore, highly stable PMA-reactive immune and mitochondrial genes suggested such differential regulation enhanced the rapid responses of this subpopulation upon reactivation (87). Since then, the combination of double positive (referred to as DP here; CD39^+^CD103^+^) has been well accepted by more researchers. For example, analysis of the aggregation of TSTs in metastatic lesions of patients with CRC after chemotherapy was performed based on the DP CD8^+^ T cell subpopulation, and this reaffirmed that DP CD8^+^ T cells were a cell subpopulation capable of reacting to mutant antigens in primary and metastatic tumors (88). In a phase Ib clinical trial (NCT02274155), researchers applied an anti-OX40 (a costimulatory molecule) agonist antibody (MEDI6469) as an adjuvant therapy prior to surgical resection in 16 patients with HNSCC and evaluated the phenotype of patients’ peripheral blood lymphocytes 2 weeks after administration. The results revealed increased activation (ICOS and CD38) and proliferative capacity (Ki67) in the CD4^+^ and CD8^+^ T cells. Tumor biopsies pre- and post-treatment showed increased frequency of activated conventional CD4^+^ TILs in the majority of patients, which was accompanied by higher oligoclonality in TCRβ sequencing. CD8^+^ TIL analysis revealed elevated proliferative capacity, and activation as well as the maintenance of the ability to identify tumor antigens specifically of tumor-specific DP T cells in patients (N=4/16) with evaluable tumor tissues, and all patients stayed disease-free (89).

### CD39^+^CD103^+^PD-1^+^CD8^+^ T cells

The results of a study evaluating single cell characteristics of TILs in human high-grade plasmacytoid ovarian cancer revealed that triple-positive (CD39^+^CD103^+^PD-1^+^) CD8^+^ TILs exhibited a highly activating/exhausted phenotype and reduced TCR diversity, and genes involved in lysis cytotoxicity and humoral immunity by comparing the cellular phenotype, clonality and prognostic significance of various combinations of three markers (CD39, CD103 and PD-1). Triple-positive CD8^+^ TILs could upregulate the expression of the cytokine CXCL13 to generate a tumor microenvironment adapted to coordinate antitumor B-cell responses. Compared with CD39^+^CD103^+^ TILs, PD-1 in triple-positive CD8^+^ TILs was associated with a higher tumor responsiveness and had the most positive impact on good patient prognosis (90). Therefore, it is necessary to define TILs with high tumor responsiveness by combining the three markers to obtain better prognostic significance.

### PD-1^+^CD137^+^CD8^+^ T cells

Recent *in vitro* tumor specificity analysis of PD-1^high^CD8^+^ T cells in ovarian cancer tissues revealed a higher frequency of CD137^+^ T cells in the cell population, despite the low quantity of this subpopulation (91). CD137, a costimulatory molecule in CD8^+^ TILs in hepatocellular carcinoma tissues, exhibited exclusive expression in highly exhausted PD-1^high^CD8^+^ T cells. CD137^+^PD-1^high^CD8^+^ T cells exhibited higher levels of tumor reactivity and T cell activation markers, and were markedly enriched in T cell inflammatory genetic features, including active IFN-γ responses, cytotoxic effector functions and activated cytokines associated with anti-PD-1 treatment responses (92). Ye (75) et al. showed directly that ovarian cancer TSTs existed exclusively in the PD-1^+^CD137^+^CD8^+^ T cell subpopulation rather than the PD-1^+^CD8^+^ T cells or PD-1−CD137^+^CD8^+^ T cell subpopulation. However, this result is inconsistent with a study of melanoma TILs, which concluded that TSTs existed in both cell populations (16). Therefore, the use of PD-1 as a single marker to screen for TSTs may be controversial and it is not possible to identify universally applicable markers due to differences in tumor types, whereas PD-1^+^CD137^+^CD8^+^ T cells are supposed to be a highly tumor-specific responsive T cell population.

### PD-1^+^ICOS^+^CD4^+^ T cells

Alspach (93) et al. demonstrated that CD4^+^ Th cells positive for PD-1 and ICOS were neoantigen-specific in a murine sarcoma tumor model. Recently, when analyzing the expression patterns of PD-1 and ICOS on CD4^+^ Th TILs in HNSCC and CRC tissues, researchers found that PD-1^+^ICOS^+^CD4^+^ Th TILs exhibited a tissue-resident memory phenotype with an oligoclonal expansion of their TCR repertoire, which occurred in tumors but was present at a low frequency in the periphery. Finally, PD-1^+^ICOS^+^CD4^+^ Th TILs were demonstrated to recognize both tumor-associated antigens and tumor-specific neoantigens (94).

## Other potential markers

### T cell factor 1 ^+^PD-1^+^CD8^+^ T cells

Tcf1 is an intranuclear transcription factor expressed in stem-like CD8^+^ T cells. Tcf1^+^CD8^+^ T cells show a highly proliferative and differentiation potential and are recognized as the most self-renewing population in TILs until now. PD-1 has been identified as a specific molecule that is upregulated on the surface of tumor reactive T cells. The results of a melanoma study identified Tcf1^+^PD-1^+^CD8^+^ T cells as a cluster of tumor reactive TILs with an exhausted phenotype and central memory characteristics by combining the aforementioned two molecules(Tcf1 and PD-1), suggesting that immune checkpoint blockade therapy results not in the functional recovery of highly exhausted cells but in promoting the strong proliferation of Tcf1^+^PD-1^+^CD8^+^ T cells to produce antitumor effects (95). Therefore, future efforts should be devoted to improving the approach to expand this subpopulation during *ex vivo* rapid expansion to enhance the potential of personalized TIL-based ACT. To further isolate and expand this subset of specific T cells with durable differentiation *in vitro*, researchers have focused on screening for cell surface markers co-expressed with Tcf1 transcription factors in different tumor types for further exploration. Previous studies have identified surface markers such as C-X-C motif chemokine receptor 5, CD28, Slamf6 (Ly108) and C-C motif chemokine receptor 4 co-expressed with Tcf1 in TILs of gastric, lung, kidney, colorectal, liver and ovarian cancer to screen for stem-like exhausted (Tcf1^+^PD-1^+^) CD8^+^ T cells to some extent. A positive association between this cell cluster and the persistent and durable effectiveness of ACT in patients has also been demonstrated (96–101). In 2020, an article published in Science reported that complete and durable control of MM requires infusion of tumor-specific CD8^+^T cells with stem cell-like profiles, which were mainly CD39−CD69−CD8^+^ T cells that co-expressed Tcf1 (102). Therefore, the innovative finding may contribute to the long-term effectiveness of personalized TIL-based ACT in oncology patients.

### PD-1^+^Bio^+^CD8^+^ T cells

Liu (103) et al. recently developed the first inter-cellular interaction glycosyltransferase-mediated labeling approach (referred to here as the FucoID strategy), where glycosyltransferase-induced transfer of a fucosylatedbiotin (Fuc-Bio)-based tag to the surface of T cells interacting with DCs accurately identified a TSA-reactive cell population in TILs in mouse tumor models, and this cell population (i.e., PD-1^+^Bio^+^CD8^+^ T cells) with a dysfunctional phenotype (almost all expressing PD-1) also showed marked proliferative and tumor-killing capacity, while a subset of PD-1^+^Bio^+^CD8^+^ T cells (4-18%) had a stem cell-like exhausted phenotype (Tcf1^+^TIM-3−). In addition, they identified a novel population of bystander T cells (PD-1^+^Bio− T cells) with completely different transcriptional characteristics compared with the previously defined bystander T cells (PD-1−CD8^+^T cells), which demonstrated that not all PD-1^+^ TILs are TSA-responsive T cells. The PD-1^+^Bio^+^CD8^+^ T cells can be directly enriched for expansion and the corresponding TCRs can be isolated to construct TCR-engineered T cells for functional assays. Thus, the FucoID strategy represents genetically manipulation-free protocols and rapid turnaround cycles compared with techniques that depend on bioinformatics-assisted TSA identification.

## Conclusions and prospects

TSTs have been demonstrated to inhibit tumor progression effectively in several clinical applications. To better sort out the cluster of T cells, researchers profit from the following observations. Firstly, some phenotypes are activated and expressed by T cells undergoing tumor antigen recognition, such as PD-1, CD39, CD103 and CD137, which have been demonstrated to be markers associated with TSTs; secondly, considering tumor reactivity and tumor responsiveness (secretion of antitumor cytokines, release of cytotoxic granules and upregulation of activation markers), some reports suggested that the combination of intracellular CD137-specific activation marker and cytokines, including IFN-γ and TNF-α, could identify tumor-specific responsive T cells more accurately (80). However, the application of TSTs defined by the aforementioned markers has exposed numerous drawbacks: The cells are mostly in a state of high exhausted and impaired effector function, the population still includes some bystander T cells and misses a low proportion of the TSA-specific effector T cell population. Extensive papers have identified a cluster of stem-cell like exhausted T cells in TILs, the main marker of which is the transcription factor Tcf1, with the combination of tumor responsive surface molecule PD-1 can recognize a population of TILs with sustained proliferative and differentiation potential. The high proliferative activity, high differentiation potential and dysfunctional T cells can produce durable tumor suppressive ability after *in vivo* transfusion. Furthermore, de Vries (104) et al. revealed immune characteristics of local tumor tissues and the systemic environment in patients with CRC by high-dimensional analysis and identified a population of innate lymphocytes (ILCs) with the exclusion of CD4^+^/CD8^+^ T cells. ILCs were defined as Lineage(Lin)−CD7^+^CD127−CD56^+^CD45RO^+^, a population that was enriched in CRC tissues and exhibited marked antitumor activity with a tumor-resident profile (CD103^+^CD69^+^). The results also indicated the diversity of tumor-specific cell populations, which should be enriched and sorted out as much as possible to achieve optimal therapeutic effects.

As all aforementioned potential biomarkers, a single marker may not meet the need for accurate sorting of specific TILs. Therefore, combined markers may be more valuable for application. However, different tumor types have different TIL components, and the tumor immune microenvironment may be the crucial factor affecting the ratio of TSTs to bystander T cells in TILs. It increases challenges to sort out the most specific tumor reactive T cells in different tumor types accurately. In the present review, it is necessary to summarize the biomarkers of TILs from different tumor types (**Table 3**). Furthermore, moving beyond these empirical cell phenotypes, the FucoID strategies that employ biochemical labeling and capture tumor antigen-specific T cells hold more promise for clinical applications. In addition, the straightforward approach to estimate TIL enrichment with tumor responsive clones based on the TCR repertoire, at the R&D phase, could greatly facilitate the selection of optimal TIL subsets and the optimization of TIL culture conditions and downstream enrichment procedures, and in clinical applications, it should be possible to estimate tumor-specific TIL abundance at the level of independent tumor samples, before and after culture and/or enrichment (84, 105, 106). In the future, the precise isolation of TSTs from different tumor microenvironments remains a major challenge to be overcome. The combination of other therapeutic approaches to further enhance the antitumor capacity of sorted TSTs, such as adding specific agonist antibodies (4-1BB, OX40, etc.), immune checkpoint inhibitors (PD-1, CTLA-4, etc.) and related cytokines (IL-2, IL-15, etc.), provides advantages for clinical application. In conclusion, TSTs remain the most active antitumor cell component in the clinical application of personalized TIL-based ACT and there is a great necessity for them to receive close attention.

**Table 3:** Potential biomarkers for the identification of tumor specific T cell in human cancers.

Tumor types	Biomarkers	Tissue of origin	Stage of disease	Reference
Melanoma	PD-1^+^	Tumor and Peripheral blood	Stage IV;	(16, 21, 22, 26, 27)
	CD39^+^	Tumor and Peripheral blood	Stage IIIC-IVB;	(42, 80, 88, 93)

	CD103^+^	Tumor	Stage I-IV;	(61)
	CD137^+^	Tumor	Stage IV;	(17, 75)
	CD39^+^PD-1^+^	Tumor	Stage IV;	(83)
	CD39^+^CD103^+^	Tumor	N/A	(19)
	CD137^+^TNF^+^IFN-γ^+^	Tumor	Stage IV;	(79)
	Tcf1^+^PD-1^+^	Tumor	N/A	(100, 101)
Ovarian Cancer	CD137^+^	Tumor	N/A	(77)
	CD39^+^PD-1^+^	Tumor	Stage I-IV;	(82)
	CD39^+^CD103^+^	Tumor	N/A	(19)
	CD39^+^CD103^+^PD-1^+^	Tumor	Stage I-IV;	(89)
	PD-1^+^CD137^+^	Tumor	Stage IA-IIIC;	(74, 90)
Non-Small Cell Lung Cancer	CD39^+^	Tumor	Stage I-IV;	(7, 40)
	CD103^+^	Tumor	Stage I-IVB;	(50, 51, 56, 60, 62–64)
	CD39^+^CD103^+^	Tumor	N/A	(19)
	Tcf1^+^PD-1^+^	Tumor	Stage I-II;	(96)
Colorectal Cancer	PD-1^+^	Tumor	Stage IIA-IVA;	(23)
	CD39^+^	Tumor	Stage I-IV;	(7)
	CD103^+^	Tumor	N/A	(19)
	CD39^+^CD103^+^	Tumor	Stage I-IV;	(85, 87)
	PD-1^+^ICOS^+^	Tumor	N/A	(93)
Hepatocellular Carcinoma	CD39^+^	Tumor	BCLC stage A-C;	(35, 36)
	CD103^+^	Tumor	Stage I-IV;	(55, 78)
	PD-1^+^CD137^+^	Tumor	N/A	(91)
	Tcf1^+^PD-1^+^	Tumor	Stage II-III;	(98)
Breast Cancer	PD-1^+^	Tumor	Stage 0-IIB;	(23)
	CD39^+^	Tumor	Stage 0-IV;	(41)
	CD103^+^	Tumor	Stage I-IV;	(52, 54)
Gastrointestinal Cancers*	PD-1^+^	Peripheral blood	N/A	(28)
Gastric Cancer	Tcf1^+^PD-1^+^	Tumor	Stage II-III;	(95)
Renal Cell Carcinoma	PD-1^+^	Tumor and Peripheral blood	Stage IV;	(27)
	CD137^+^	Tumor	Fuhrman grade I-IV;	(80)
	Tcf1^+^PD-1^+^	Tumor	Stage I-IV;	(97)
Cervical Cancer	CD39^+^	Tumor	N/A	(38)
	CD103^+^	Tumor	Stage IA2-IVA;	(49)
	CD39^+^PD-1^+^	Tumor	N/A	(84)
Vulvar Squamous Cell Carcinoma	CD39^+^	Tumor	N/A	(38)
Oropharyngeal Squamous Cell Carcinoma	CD39^+^	Tumor	N/A	(38)
	CD103^+^	Tumor	Stage I-III;	(57)
Endometrial Adenocarcinoma	CD103^+^	Tumor	FIGO stage I-IV;	(53)
	CD39^+^PD-1^+^	Tumor	FIGO stage I-IV;	(82)
	CD39^+^CD103^+^	Tumor	FIGO stage IA-IIIC;	(86)
Esophageal Squamous Cell Carcinoma	CD103^+^	Tumor	Stage I-III;	(18)
Head and Neck Squamous Cell Carcinoma	CD39^+^PD-1^+^	Tumor	N/A	(84)
	CD39^+^CD103^+^	Tumor	Stage II-IVA;	(19, 88)
	PD-1^+^ICOS^+^	Tumor	N/A	(93)
Urothelial Carcinoma	CD103^+^	Tumor	Stage I;	(62)
Bladder Cancer	CD39^+^	Tumor	Stage I-IV;	(37)
	CD103^+^	Tumor	Stage I-IV;	(47)

*Gastrointestinal Cancers including pancreatic、gastroesophageal、colon and bile duct cancer. N/A, Not Applicable.

## Author contributions

WG performed the analyses and wrote the manuscript. YQD, YD, LC contributed to the conception of the paper. JC, ML, JW contributed to analysis. WW, XM helped perform the analysis with constructive discussions. All authors contributed to the article and approved the submitted version.

## Funding

This project was supported by grants from the National key research and development program (Grant No: 2019YFA0110703),Science and Technology Innovation Foundation of Hunan Province (Grant No: 2020SK53614) and Natural Science Foundation of Hunan Province, China (Grant No: 2021JJ31018; 2020JJ4841) .

## Conflict of interest

The authors declare that the research was conducted in the absence of any commercial or financial relationships that could be construed as a potential conflict of interest.

The reviewer QC declared a shared affiliation, with no collaboration, with the authors to the handling editor at the time of the review.

## Publisher’s note

All claims expressed in this article are solely those of the authors and do not necessarily represent those of their affiliated organizations, or those of the publisher, the editors and the reviewers. Any product that may be evaluated in this article, or claim that may be made by its manufacturer, is not guaranteed or endorsed by the publisher.
